# The Discovery Research Platform for Medical Humanities responds to the Wellcome Trust’s report on *Archives, manuscripts and material culture (AMCs) in life, health, and wellbeing research*

**DOI:** 10.12688/wellcomeopenres.25676.1

**Published:** 2026-02-14

**Authors:** Harriet Barratt, Fiona Johnstone, Coreen McGuire

**Affiliations:** 1 Discovery Research Platform for Medical Humanities, Institute for Medical Humanities, Durham University, Durham, England, UK; 2 School of Modern Languages & Cultures, Durham University, Durham, England, UK; 3 Durham University Department of History, Durham, England, UK

**Keywords:** Medical humanities; material culture; history; archives; access and inclusivity; curating; artificial intelligence; disability history

## Abstract

This open letter from Durham University’s Discovery Research Platform for Medical Humanities responds to the Wellcome Trust’s 2025 report on archives, manuscripts, and material culture (AMCs) in health research. We applaud the report’s recognition of AMCs as foundational infrastructures for discovery research and its emphasis on pluralistic values, and we use this letter to expand upon three critical dimensions. First, we reinforce the point that medical humanities work with AMCs generates transformational biomedical and cultural knowledge capable of effecting real-world reform, exemplified by recent challenges to race-based lung capacity evaluations citing Platform research. Second, we urge caution regarding digitisation initiatives, particularly concerning A.I. training using existing collections, emphasising the need for ethical consideration of environmental impact, intellectual property, and the perpetuation of structural inequities in digital infrastructure. Third, we advocate for expansive definitions of health-related material culture beyond biomedical paradigms, encompassing artistic collections and emphasising the irreplaceable value of sensory and affective experiences in hands-on collections work. We draw attention to the tensions inherent in the use of digital surrogates, which, though vital for accessibility, cannot fully replace original collections. We conclude by emphasising the Platform’s commitment to further collaborative work to transform the report’s recommendations into sustainable practice.

## Plain Language summary

The Discovery Research Platform for the Medical Humanities at Durham University received the recent Wellcome Trust report on archives, manuscripts and material culture in health research (2025) with enthusiasm. The report recognises that these sources provide essential foundations for discovery research. This recognition is overdue. We reiterate the report’s emphasis on valuing collections and research in multiple ways. We want to highlight three key points. **First, archives and collections produce new knowledge.** Medical humanities work can transform knowledge and create real-world change. For example, a 2025 New England Journal of Medicine article has called to end race-based lung capacity evaluations. It cited historical work by Platform researchers to argue for clinical reform and the American Medical Association (AMA) have now stated that their next Guides to the Evaluation of Permanent Impairment will remove race equations from lung function testing. **Second, we must approach digitisation with caution.** We must carefully consider AI use in relation to its environmental impact and intellectual property misuse. Digital systems often replicate structural inequities. Any work to revolutionise these practices must put equality at the heart of systems which try to classify knowledge. **Third, we must expand what counts as health-related material culture.** Health goes beyond medicine's visual and material cultures. We must include other forms of lived experience. Digital versions of objects and manuscripts are important for accessibility, but they differ from original collections. Hands-on work creates unique understandings. We look forward to transforming the report's recommendations into sustainable practice.

The Discovery Research Platform for Medical Humanities at the University of Durham received the recent *Archives, manuscripts and material culture in life, health and wellbeing research* report ( Wellcome Trust, 2025) with appreciation for both its generative possibilities and its recognition of the transformative value of the kinds of work we do at the Platform. The report’s recognition of AMCs as foundational infrastructures for discovery research in life, health and wellbeing is a long overdue recognition of the critical importance of these sources, their potential for enabling and shaping transformative methods and knowledges, and their current endangerment. We also welcome the repeated emphasis on the need for pluralistic articulations of the value of collections and associated research outputs.

We applaud the report’s recognition of the equal value of historical and material culture approaches in tandem. The Institute for Medical Humanities at Durham University has repeatedly demonstrated history’s potential to dismantle problematic inherited understandings and infrastructures: ‘history does more than critically break down categories, it is also crucial in building anew’ ( Bellis et al., 2024). Equally, we have long championed the potential of collections-based and object-led work for advancing knowledge about life, health and wellbeing, including via our *Thinking Through Things: Object Encounters in Medical Humanities* series (2019-21) and the international project *Curating Cultural Heritage for the Medical and Health Humanities* (2024-26). Our Visual and Material Lab, part of the Wellcome-funded Discovery Research Platform for Medical Humanities, was set up explicitly to address many of the issues and challenges raised by this report.

Here we identify **three key aspects** of the report that we wish to expand upon and, in a few areas, to raise notes of caution.

First, we wish to reinforce the point that **AMCs are epistemically and epistemologically productive**. The report contains a notably lucid articulation of the value of AMCs’ contribution to discovery research (p. 23): epistemic, contextualising, critical and reflective, participatory and reparative, and speculative and imaginative. To extend this, we want to re-emphasise that medical humanities work with AMCs can create *new* biomedical and cultural knowledge. Indeed, the claim that medical humanities work can *create* transformational knowledge as well as critique inherited understandings — and that it can effect real-world change — has been the cornerstone insight motivating the development of critical medical humanities over the last ten years ( Viney et al., 2015). As such, while the examples provided here highlight productive archival practices (p. 23), we call for an ambitious shared vision supporting novel approaches that goes beyond traditional research methods and applications to advance knowledge and actively reshape policy. As a brief example: in 2025 *The New England Journal of Medicine* called for an end to race-based evaluations of lung capacity which have been inappropriately used in occupational disability compensation ( Khazanchi et al., 2025). This article cited archival research and advocacy conducted by Platform researchers as a basis from which to argue for clinical reform and remedy these historical harms. The American Medical Association (AMA) have now stated that their next *Guides to the Evaluation of Permanent Impairment* will remove race equations from lung function testing.

Second, we note that the report’s reflection on the possibilities of digitisation were suitably nuanced; ‘digitisation remains a double-edged sword’ (p. 37). However, we emphasise that **caution is paramount when considering the digitisation of existing resources** and that ethical consideration must be given to the use of A.I., its environmental impact, and its problematic use of intellectual property. The use of existing collections to train generative models was discussed relatively uncritically in the report (p. 27), despite the recognition (p. 43) that digital infrastructure and collections alike often replicate structural inequities and silence particular voices ( Veenhuizen and O’Malley, 2025). Any work to revolutionise these practices must centralise and innovate ways of working equitably with systems of knowledge classification, and actively address engineered bias. Concurrently, it is worth bearing in mind that if these classification systems are themselves historical products, then they are themselves worth preserving (though not perpetuating) in some form. For instance, disability historians have developed methodologies to recover disabled voices in hostile institutional archives that were never designed to amplify their voices. Material objects have been similarly critical to this endeavour; ‘disability history remains bound to its focus on the material’ ( McGuire, 2024). This insight brings us to our third and final point, which concerns the categorisation of material culture.

Our final point concerns **classifications of ‘what counts’ as the material culture of health and wellbeing, and the importance of sensory and affective experience within its study**. Collections-led medical humanities research at Durham has long taken the view that health cannot be comprehended through conventional understandings of the visual and material cultures of medicine alone, but must encompass other forms of lived and embodied experience. As such, we were heartened by the report’s call for ‘a more expansive sense of what counts as a “health-related” collection’ to unlock perspectives ‘that extend far beyond biomedical paradigms’ (p. 32). This includes artistic collections (a form of material culture in their own right and a curious omission from the report’s landscape mapping) and, at the other end of the scale, micro-molecular innovations that increasingly challenge our preconceptions of what may count as a conventional, tangible heritage object ( Söderqvist et al., 2009). We welcome the aspiration to ‘value the interpretive work of researchers and curators alike’ in funding and institutional frameworks, offering exciting potential to embed curatorial practice more centrally into primary research and engagement, and to further explore what visual and material artefacts ‘can do for us’ clinically, personally and socio-culturally ( Johnstone, 2018). This includes the potential for collections, research and curatorship in tandem to further social justice and deliver forms of social care ( Tolia-Kelly et al., 2016; Morse, 2020). As a note of caution, we draw attention to the tension inherent within the use of digital surrogates (for example, online catalogues or 3D replicas). While vitally important for inclusive accessibility, we must retain a careful awareness that they differ from original collections in form, function and use. As scholars from the fields of museology and sensory studies have repeatedly demonstrated ( Dudley, 2010; Smith and Campbell, 2015; Howes, 2022), hands-on collections work elicits understandings and analyses not otherwise available to researchers, and investment in equitable in-person collections access must not be de-prioritised within digitisation initiatives.

In summary, we warmly welcome this insightful and innovative report. We look forward to working critically and collaboratively with a broad range of stakeholders to transform its ambitious recommendations into sustainable practice.

## Disclaimer

The views expressed in this article are those of the authors. Publication in Wellcome Open Research does not imply endorsement by Wellcome.

## Data Availability

No data are associated with this article.
