# Supplementary material for: The Discovery Research Platform for Medical Humanities responds to the Wellcome Trust’s report on Archives, manuscripts and material culture (AMCs) in life, health, and wellbeing research
Source: Wellcome Open Res. 2026 Feb 14;11:111. [Version 1] doi: 10.12688/wellcomeopenres.25676.1 (PMC12949836; doi:10.12688/wellcomeopenres.25676.1)
Supplement: Peer review history

# Reviewer report: Reviewer response for version 1

**DOI:** 10.21956/wellcomeopenres.28274.r149197

**Published:** 2026-03-12

**Authors:** Christine Slobogin

**Affiliations:** 1 University of Rochester, Rochester, New York, USA

This Open Letter, responding to the Wellcome Trust report on archives, manuscripts, and material culture in health research (2025), makes three key points. While these statements are not overwhelmingly critical of the report, they do raise good points about where and how the report could have been more nuanced or shown more caution. The publication of these areas of caution is useful for those of us who read the report and who want to make sure that the Wellcome's word on AMCs in health research is not the final word on what these resources can, or should, do.

 I particularly welcome the authors' statement "that caution is paramount when considering the digitization of existing resources and that ethical consideration must be given to the use of A.I., its environmental impact, and its problematic use of intellectual property." The medical humanities is at its best when it integrates ethical discussion - and the ethics of AI is vital to consider in both historical and health research. A further reflection on AI in the Wellcome report (how it was used in the report itself, and how it can or should be used in future work with AMCs) would have been welcome, and I am glad that the authors of this Open Letter bring it up.

 I recognize that the authors are writing explicitly from the perspective of being part of the Discovery Research Platform for Medical Humanities at the University of Durham - otherwise this Open Letter would have likely been too long and convoluted. But one space for continued thinking on and expansion here would be the inclusion of work of those at other institutions who are also proving the authors' points. By calling out this work explicitly in the Open Letter, the authors could have made this response feel less siloed to just their own institution.

Does the article adequately reference differing views and opinions?

Yes

Are all factual statements correct, and are statements and arguments made adequately supported by citations?

Yes

Is the Open Letter written in accessible language?

Yes

Where applicable, are recommendations and next steps explained clearly for others to follow?

Yes

Is the rationale for the Open Letter provided in sufficient detail?

Yes

Reviewer Expertise:

visual medical humanities, art history

I confirm that I have read this submission and believe that I have an appropriate level of expertise to confirm that it is of an acceptable scientific standard.

# Reviewer report: Reviewer response for version 1

**DOI:** 10.21956/wellcomeopenres.28274.r149191

**Published:** 2026-03-10

**Authors:** Bushra Juhi Jani

**Affiliations:** 1 Al-Nahrain University, Baghdad, Iraq

This Open Letter provides a thoughtful and well-argued response to the Wellcome Trust’s report on archives, manuscripts, and material culture in health research. The authors effectively highlight the transformative role of medical humanities and make a persuasive case for the broader epistemic and ethical significance of archival infrastructures.

 The discussion of digitisation and artificial intelligence is particularly strong, offering necessary ethical depth and critical awareness regarding environmental impact, intellectual property, and structural inequalities. The call to expand definitions of health-related material culture beyond strictly biomedical paradigms is also conceptually valuable.

 However, the article would benefit from:

- More specific engagement with particular sections or recommendations from the Wellcome report.
- Clearer evidentiary framing of the example concerning race-based lung capacity evaluations.
- Slightly more precise clarification of how digital surrogates alter research interpretation.

Overall, the piece is coherent, accessible, and makes a meaningful contribution to ongoing discussions in medical humanities and archival research.

Does the article adequately reference differing views and opinions?

Yes

Are all factual statements correct, and are statements and arguments made adequately supported by citations?

Yes

Is the Open Letter written in accessible language?

Yes

Where applicable, are recommendations and next steps explained clearly for others to follow?

Yes

Is the rationale for the Open Letter provided in sufficient detail?

Yes

Reviewer Expertise:

Medical and Health Humanities, English Literature, Arabic Literature, African Literature, Translation Studies, Film Studies

I confirm that I have read this submission and believe that I have an appropriate level of expertise to confirm that it is of an acceptable scientific standard.

# Reviewer report: Reviewer response for version 1

**DOI:** 10.21956/wellcomeopenres.28274.r149190

**Published:** 2026-02-28

**Authors:** Valerio Ferro Allodola

**Affiliations:** 1 Mediterranea University of ​​Reggio Calabria, Calabria, Italy

The article provides a robust and well-argued response to the Wellcome Trust’s 2025 report on archives, manuscripts and material culture (AMCs) in life, health and wellbeing research. The contribution is persuasive, theoretically sound, and well positioned within current debates in critical medical humanities.

**Main strengths:**

 - It convincingly highlights the epistemic productivity of AMCs, demonstrating that medical humanities not only offer critique but also generate transformative knowledge with tangible clinical and policy impact.

 - It offers a balanced reflection on digitisation and AI, foregrounding ethical, environmental and structural inequality concerns.

 - It broadens the notion of material culture in health by incorporating artistic, sensory and embodied dimensions, and defends the importance of direct, in-person access to collections.

**Areas for improvement:**

 - Reduce the emphasis on institution-specific initiatives in order to strengthen the sector-wide perspective.

 - Provide greater conceptual clarity around the notion of “transformational knowledge”.

 - Make the practical implications and policy recommendations more explicit.

**Overall assessment:**

 A significant, mature and publishable contribution, and of clear interest to scholars, policy-makers and research funders in the fields of medical humanities and research infrastructure.

Does the article adequately reference differing views and opinions?

Yes

Are all factual statements correct, and are statements and arguments made adequately supported by citations?

Yes

Is the Open Letter written in accessible language?

Yes

Where applicable, are recommendations and next steps explained clearly for others to follow?

Yes

Is the rationale for the Open Letter provided in sufficient detail?

Yes

Reviewer Expertise:

Medical Humanities and Narrative Based Medicine

I confirm that I have read this submission and believe that I have an appropriate level of expertise to confirm that it is of an acceptable scientific standard.

# Reviewer report: Reviewer response for version 1

**DOI:** 10.21956/wellcomeopenres.28274.r148320

**Published:** 2026-02-23

**Authors:** Chase Ledin

**Affiliations:** 1 University of Edinburgh, Edinburgh, UK

The open letter responds to the Wellcome Trust's 2025 report on archives, manuscripts and material culture in health research. The letter is clear, purposeful and contains a good balance of positive support and constructive criticism. The authors consider important gaps where further research may be undertaken and/or funded by Wellcome. This open letter is sufficiently cited and accurately presents the authors' position(s) and experience(s) at IMH Durham.

Does the article adequately reference differing views and opinions?

Yes

Are all factual statements correct, and are statements and arguments made adequately supported by citations?

Yes

Is the Open Letter written in accessible language?

Yes

Where applicable, are recommendations and next steps explained clearly for others to follow?

Not applicable

Is the rationale for the Open Letter provided in sufficient detail?

Yes

Reviewer Expertise:

Medical sociology, public health, science and technology studies, medical humanities, science studies, queer studies

I confirm that I have read this submission and believe that I have an appropriate level of expertise to confirm that it is of an acceptable scientific standard.
